# A Complete Genome of the Alphaproteobacterial Methanotroph Methylocystis parvus OBBP

**DOI:** 10.1128/mra.00076-23

**Published:** 2023-03-23

**Authors:** Benedict H. Claxton Stevens, Bashir L. Rumah, Christopher E. Stead, Ying Zhang

**Affiliations:** a BBSRC/EPSRC Synthetic Biology Research Centre, School of Life Sciences, Biodiscovery Institute, University of Nottingham, Nottingham, United Kingdom; Wellesley College

## Abstract

A complete genome is presented for Methylocystis parvus OBBP, a Gram-negative aerobic methanotroph of the phylum *Alphaproteobacteria*. *M. parvus* OBBP is the genus type strain and of interest in the production of polyhydroxybutyrate and environmental microbiology. The genome consists of two plasmids (248 kbp and 205 kbp) and a chromosome (4.076 Mbp).

## ANNOUNCEMENT

Methylocystis parvus OBBP is an obligate methanotroph of interest in the production of the biodegradable bioplastic polyhydroxybutyrate (PHB) and is the genus type strain of *Methylocystis* (1–3). An unclosed *M. parvus* OBBP genome (MetPar_1.0-AJTV00000000.1) of 108 contigs has been previously released (1), which has been widely used, including a genome-scale model in 2019 (4). As an improvement upon this, sequencing and error checking of the new genome were exhaustive to ensure a complete and reliable reference was achieved.

Strain OBBP, isolated in 1970 from soil and water samples (2), was acquired from NCIMB 11129 (National Collection of Industrial, Food and Marine Bacteria). OBBP was grown in pure culture in nitrate mineral salt (NMS) medium (2) at 30°C, and genomic DNA (gDNA) was extracted using a phenol-chloroform-isoamyl alcohol method (5). A library of sheared large insert gDNA was prepared using g-TUBES (Covaris, Woburn, MA, USA) at 4,000 rpm once per end for 60 s and sequenced without size selection using HiFi PacBio on an RS II SMRTcell by Genome Quebec, Canada. The PacBio SMRTPipe pipeline collapsed and error corrected the reads, giving 145,723 ~10-kbp circular consensus sequencing reads totaling 1,333,680,793 bp, an *N*_50_ of 9,860 bp, and 293× depth. Short-read gDNA libraries were prepared using the Nextera XT library prep kit (Illumina, San Diego, CA, USA) and processed on an Illumina MiSeq (Deep Seq, Nottingham, UK) using a 250-bp paired-end protocol, resulting in 987,357 paired reads totaling 480,751,157 bp and 104× depth. Quality of reads was checked using FastQC (v0.11.9) (6).

A hybrid assembly strategy was followed. PacBio reads were assembled with SMRTLink (v8.0.0.80501), and PacBio and Illumina reads were assembled together in Unicycler (v0.4.9) (7), providing two assemblies. Realignment was carried out with Bowtie2 (v2.4.4) (8) for short reads and minimap2 (v2.24) (9) for long reads with iterated polishing with Pilon (v1.24) (10). Assemblies were compared with Mauve (v20150226) (11) and ALE (v0.9) (12) and visualized with IGV (v2.11.9) (13). Both assemblies informed the final genome. Discrepancy decisions were confirmed by Sanger sequencing of PCR products by Genewiz, USA.

The final genome (Table 1) consists of a chromosome and two plasmids (pMpar-1 and pMpar-2) with no gaps or undecided bases. The genome achieved a BUSCO (v5.3.0) (14) completeness of 99.6% against the v10 *Alphaproteobacteria* data set and a CheckM (v1.1.3) (15) score of 100%.

**TABLE 1:** Assembly and annotation summary

Parameter	Total	Chromosome	pMpar-1	pMpar-2
GenBank accession no.		CP092968	CP092969	CP092970
Size, bp	4,529,117	4,076,007	248,224	204,886
GC, %	63.39	63.7	60.8	60.3
Genes, no.	4,379	3,923	226	230
Hypothetical genes, no. (%)	823 (18.8)	702 (17.9)	59 (26.1)	62 (27.0)

Annotation was carried out using the NCBI Prokaryotic Genome Annotation Pipeline (PGAP, v6.0) (16) and summarized in Table 1. Of these annotations, 93 were pseudogenes. Annotations included two full sets of rRNAs, 48 tRNAs, and two CRISPR arrays. The new genome adds 57.3 kbp to the MetPar_1.0 assembly including six additional *pmo* genes and removes nine unplaced contaminant contigs: six Priestia megaterium, one Stenotrophomonas rhizophila, one *Mesorhizobium* sp., and one Pseudomonas putida.

### Data availability.

This genome has been deposited in GenBank as CP092968 (chromosome), CP092969 (pMpar-1), and CP092970 (pMpar-2). The version described in this paper is the first version. Sequencing data are under the NCBI BioProject accession no. PRJNA812408 containing Illumina, PacBio, and Sanger sequencing data with additional methylation data.
